# Extraction and Characterization of Cellulose from Jerusalem Artichoke Residue and Its Application in Blueberry Preservation

**DOI:** 10.3390/foods11081065

**Published:** 2022-04-07

**Authors:** Xiaotong Zhou, Liu Liu, Jianke Li, Lin Wang, Xueying Song

**Affiliations:** 1 College of Food Engineering and Nutritional Science, Shaanxi Normal University, Xi’an 710119, China; auty_zhou@126.com (X.Z.); jiankel@snnu.edu.cn (J.L.); wangliner417@163.com (L.W.); sxy18734445184@163.com (X.S.); 2 Key Laboratory of Food Processing Byproducts for Advanced Development and High Value Utilization, Shaanxi Normal University, Xi’an 710119, China

**Keywords:** Jerusalem artichoke, soluble dietary fiber, blueberry

## Abstract

The utilization of industrial by-products is becoming more and more important for resource utilization. In this study, soluble dietary fiber (SDF) was extracted from Jerusalem artichoke residue, and a series of characterizations of SDF were carried out. The results showed that SDF had good properties. SDF (0%, 0.1%, 0.2%, 0.3%, and 0.4%) and chitosan (2%) were further used to prepare the coating that was used for the preservation of blueberry. The chemical structure of the film was obtained by FT-IR and XRD analysis. The microstructure of the film was analyzed by SEM, and the properties of the film were tested. The blueberry fresh-keeping test proved that the SDF-added film could effectively prolong the quality of blueberries in storage for 16 days. After 16 days of storage, compared with the control group, the decay rate of the coating group with 0.2% SDF decreased by 16.3%, the consumption of organic acids decreased by 43.7%, and the content of anthocyanin increased by 29.3%. SDF has a potential application in food preservation.

## 1. Introduction

The Jerusalem artichoke (*Helianthus tuberosus* L.) is a perennial sunflower herb in the Compositae family. It was introduced into China from Europe and is widely planted in north China. The Jerusalem artichoke is a potential energy crop with a high yield and rich nutrients, especially inulin [1]. Seeing its excellent physiological functions and wide application in food, the inulin industry has developed rapidly in recent years. Jerusalem artichoke residue is a by-product of inulin processing, which contains starch, cellulose, high quality protein, and other important available resources. Jerusalem artichoke residue is basically fed to livestock as a feed after drying, mostly without nutritional optimization. In addition, most Jerusalem artichoke residue is discarded as waste. This way has caused environmental pollution and a great waste of resources. At present, the research on Jerusalem artichoke and Jerusalem artichoke residues mainly focuses on the preparation of silage. For example, a study carried out by Piotr Koczocine et al. [2] studied the chemical changes that occur in Jerusalem artichoke silage. There are few reports on the comprehensive utilization of favorable components in Jerusalem artichoke residue.

Cellulose is a macromolecular polysaccharide composed of glucose. Dietary fiber is the general term for polysaccharides that cannot be digested by human body in food, which is divided into soluble dietary fiber and insoluble dietary fiber. Most dietary fibers are structural polysaccharides in plant cell walls. More and more studies have shown that dietary fiber has important physiological functions in the human body, especially in the human intestinal tract. In addition, based on the characteristics of dimensions, crystallinity, surface charge, and wettability, cellulose has excellent foaming, emulsifying, and film-forming ability. Therefore, cellulose is also widely used in food additives and has a high development and utilization value [3]. The preparation methods of cellulose are mainly chemical separation or a combination of chemical reagents and enzymes. The raw materials for preparation are wheat bran, corn bran, tofu residue, etc. [4]. In this study, soluble dietary fiber (SDF) was extracted from Jerusalem artichoke residue by a hydrothermal method.

Blueberries (*Vaccinium* ssp.) are a species from the family Ericaceae, which have high nutritional value and are rich in bioactive substances, such as vitamins, anthocyanins, and other phenolic compounds [5]. Fresh blueberry skin is thin and juicy and is vulnerable to mechanical damage and microbial infection, affecting post-harvest storage, transportation, and sales. As the consumption of blueberries increased year by year, the problems in storage appeared. Blueberries are easily spoiled by water loss and mold growth. In order to minimize the deterioration of blueberries, factors affecting the initial quality (variety and maturity) of products and subsequent treatment methods should be considered. After harvest, storage temperature, humidity, and atmosphere are key to preventing early decay and prolonging the shelf life of blueberries. In addition, blueberry preservation strategies include chemical strategies, physical strategies, and biological strategies. Studies have shown that blueberries can be successfully stored at low temperatures (0–5 °C) for two to seven weeks. This study is expected to extend the storage period of blueberry at room temperature [6].

Coating technology is often used in fruit preservation research. Marina Paolucci et al. [7]. studied active edible polysaccharide-based coating for the preservation of fresh figs (*Ficus carica* L.). Bing Xie et al. [8]. investigated an edible coating based on a beeswax-in-water Pickering emulsion stabilized by cellulose nanofibrils and carboxymethyl chitosan. This edible coating has a good application prospect in berry preservation. Chitosan has certain antibacterial effects and is a good substrate for the coating of fruits and vegetables. However, the use of chitosan alone for coating has limitations. In this study, SDF extracted from Jerusalem artichoke extract was used as a raw material, and chitosan was compounded to prepare an edible coating for blueberry preservation to prolong the storage period of fresh blueberries at room temperature.

In this study, SDF was extracted from Jerusalem artichoke residue, a waste product of the inulin industry. The edible preservation coating was prepared with SDF and applied to the preservation of blueberries. The goal of this work is to realize the extension of the inulin industry, develop the added value of industrial waste, and improve waste utilization.

## 2. Materials and Methods

### 2.1. Materials and Reagents

Jerusalem artichoke residue was provided by Shaanxi Sciphar Natural Products Co., Ltd. (Xi’an, Shaanxi, China). Chitosan (90%, deacetylated) was supplied by Aladdin Chemical Co. (Shanghai, China). Other chemical reagents were analytically pure.

### 2.2. Extraction of Soluble Cellulose

First, 5 g of Jerusalem artichoke residue powder was placed in a 250 mL beaker, 100 mL distilled water, 0.3 g of ammonium sulfate, and 0.325 g of potassium dihydrogen phosphate were added, and extraction was carried out at 80 °C for 1 h after decompression filtration. The combined filtrate was repeatedly extracted three times, and then all the filtrate was concentrated to a quarter of the original volume at 80 °C and cooled to room temperature. Then, four times the volume of anhydrous ethanol was added, the mixture stood for 4 h after centrifugation, and soluble dietary fiber (SDF) was collected via precipitation drying.

### 2.3. Characterization of SDF

The characterization method of SDF referred to the research of Shi Sheng et al. [9] and Bianjing Sun et al. [10] and was slightly modified.

The thermal degradation processes of the SDF were measured using a Thermoanalyzer Systems instrument (Q1000DSC + LNCS + FACS Q600SDT, TA Instruments, New Castle, DE, USA). All samples were scanned from 50 to 600 °C at a heating rate of 10 °C/min and a nitrogen flow rate of 60 mL/min.

The appropriate amount of dried SDF was mixed with KBr and then ground to prepare the tablets. The infrared spectrum was analyzed by a Nicolet iS10 spectrometer (Thermo Fisher Scientific Inc., Waltham, MA, USA) in the range of 400–4000 cm^−1^.

The SDF was analyzed by X-ray diffraction (D8 Advance, Brock, Germany). The scanning speed was 5/min, and the range of 2θ was 5–50°.

The SDF powder was fixed on the metal support with a double-sided conductive adhesive and coated with gold. The microstructures of the cellulose, such as cross-sectional and surface morphology, were characterized by SEM (FEI-Quanta 200, Hillsboro, OR, USA).

### 2.4. Preparation of Chitosan Composite Coating Solutions and Films

The chitosan composite coating solutions and films were prepared with reference to the method of Xueying Song et al. [11] and slightly modified. SDF was dissolved in 100 mL of 1% acetic acid solution according to Table 1. After the SDF was completely dissolved, 2 g of chitosan was added and stirred with a magnetic stirrer until the coating-forming solution was transparent. Then, 0.6 mL of glycerol was added as a plasticizer and stirred for 60 min. The coating solutions were degassed by an ultrasonic cleaning machine for 30 min. Then, 15 mL of coating solution was poured into a circular container with a diameter of 90 mm and dried. After drying, the film was placed in a vacuum dryer for standby.

### 2.5. Characterization of Films

#### 2.5.1. Determination of Moisture Content, Water Solubility, and Water Vapor Permeability (WVP)

The initial film weight (M1) was dried to a constant weight (M2) at 105 °C. In the water solubility determination, the dry film was placed in a beaker containing distilled water and sealed with a plastic film for 24 h. Then, the film was removed for rapid drying and dried again at 105 °C to a constant weight (M3) [12].

Each film was fixed in a glass containing anhydrous calcium chloride. After weighing, the glass was placed in the dryer and weighed daily until the mass change was less than 0.001 g. W (g): increased weight, X (m): thickness, t (s): time required to reach the constant weight, A (m^2^): penetration area, P: partial vapor pressure difference between dry atmosphere and pure water (2339 Pa at 20 °C) [13].

(1) $$\mathrm{Moisture}\;\mathrm{content}\;(\%)=\frac{M1-M2}{M1}\times 100\%$$

(2) $$\mathrm{Water}\;\mathrm{solubility}\;(\%)=\frac{M2-M3}{M2}\times 100\%$$

(3) $$\mathrm{Water}\;\mathrm{vapor}\;\mathrm{permeability}=\frac{W\;\times \;X}{t\;\times \;A\;\times \Delta P}$$

#### 2.5.2. SEM, TGA, FTIR, and XRD Analyses of the Films

The films were cut to the appropriate size, fixed on the metal support with a double-sided conductive adhesive, and coated with gold. The microstructures of the films, such as cross-sectional and surface morphology, were characterized by SEM (FEI-Quanta 200, Hillsboro, OR, USA).

The thermal degradation processes of the films were measured using a Thermoanalyzer Systems instrument (Q1000DSC + LNCS + FACS Q600SDT, TA Instruments, New Castle, DE, USA). All samples were scanned from 50 to 600 °C at a heating rate of 10 °C/min and a nitrogen flow rate of 60 mL/min.

Each film was attached to the metal support. The infrared spectrum was analyzed by a Nicolet iS10 spectrometer (Thermo Fisher Scientific Inc., Waltham, MA, USA) in the range of 400–4000 cm^−1^.

Each film was analyzed by X-ray diffraction (D8 Advance, Brock, Germany). The scanning speed was 5/min, and the range of 2θ was 5–50° [14,15].

### 2.6. Blueberry Preservation

#### 2.6.1. Application of Edible Coatings

Six groups of blueberry samples were prepared. The control group was not coated. The other five groups were immersed in chitosan coating solutions containing 0.00%, 0.10%, 0.20%, 0.30%, and 0.40% cellulose. The coating solution was evenly wrapped and dried. Each sample was stored for 16 days, and the corresponding indexes were measured every 4 days.

#### 2.6.2. Determination of Weight Loss Rate, Decay Rate, and Frost Covering Rate

The mass of the blueberries was recorded the first time (m0) and each time (mf). The weight loss rate (W) of the blueberries was calculated according to the formula:

(4) $$W\;(\%)=(\frac{m0-\mathrm{mf}}{m0})\times 100$$

The decay of blueberry fruit was mainly evaluated according to the softening degree of fruit, juice leakage caused by decay, and fungal infection. The corruption situation was divided into three levels: level 0, surface has no obvious change; level 1, obvious softening phenomenon; level 2, serious softening and juice leakage; and level 3, mold spot infection on the surface. The decay rate was calculated as follows:

(5) $$\mathrm{Decay}\;\mathrm{rate}\;(\%)=\sum \frac{\mathrm{Decay}\;\mathrm{level}\times \mathrm{Numberof}\;\mathrm{fruits}\;\mathrm{at}\;\mathrm{this}\;\mathrm{level}}{\mathrm{Highest}\;\mathrm{decay}\;\mathrm{level}\times \mathrm{Total}\;\mathrm{fruit}\;\mathrm{quantity}}\times 100\%$$

According to the coverage score, blueberries were divided into four levels: level 0, fruitless cream; level 1, fruit frost covering area 0~1/3 of the fruit; level 2, fruit frost covering area is 1/3~2/3 of the fruit; level 3, fruit frost covering area is 2/3 of the fruit; and level 4, fruit frost covers the whole fruit and the fruit frost is thicker [16].

(6) $$\mathrm{Frost}\;\mathrm{covering}\;\mathrm{index}\;(\%)=\sum \frac{\mathrm{Fruit}\;\mathrm{frost}\;\mathrm{level}\times \mathrm{Number}\;\mathrm{of}\;\mathrm{fruit}\;\mathrm{of}\;\mathrm{this}\;\mathrm{level}}{\mathrm{Highest}\;\mathrm{fruit}\;\mathrm{frost}\;\mathrm{level}\times \mathrm{Total}\;\mathrm{fruit}\;\mathrm{quantity}}\times 100\%$$

#### 2.6.3. Determination of pH, Titratable Acid, and Anthocyanin Content

A juicer was used to break up the blueberries and filter them into juice. A pH meter was used to measure the pH of the juice. The titratable acid content in the blueberries was determined by reference to Carolina Medina-Jaramillo et al. [16]. Referring to Jungmin Lee’s [17] method, the anthocyanin content in blueberries was determined by the pH differential method.

#### 2.6.4. Determination of Texture of Blueberries

A texture analyzer (stable micro system-TA.XT. PLUS, UK was used to test the peel puncture strength and average flesh firmness of blueberries. The testing parameters were as follows: displacement of 5 mm and force of 10.0 g. In addition, the test was repeated six times in each group.

### 2.7. Statistical Analysis

All measurements were repeated three times and the results were averaged. The data analysis software used for the whole experiment was Microsoft Excel 2011, SPSS Statistics 26.0 (IBM, Armonk, New York, NY, USA), and RStudio 1.3. The differences between the different treatments were determined by a one-way ANOVA. The differences were considered significantly at the 95% confidence level (*p* < 0.05).

## 3. Results

### 3.1. Characterization of SDF from Jerusalem Artichoke

**Figure 1:**
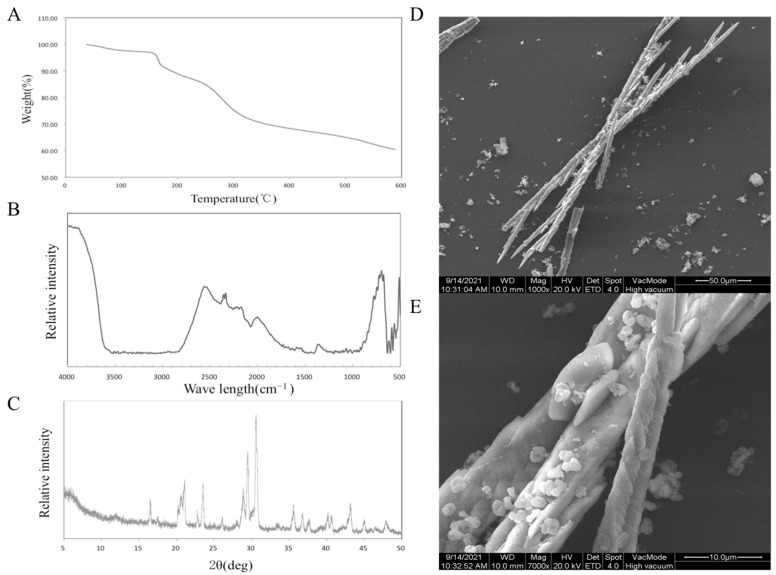
TGA analysis (**A**), FT-IR spectra (**B**), XRD patterns (**C**), SEM photo (**D**,**E**) of SDF.

Through the determination of polysaccharide content, the purity of SDF was 65%. The results of the thermal analysis are shown in Figure 1A. When the temperature is lower than 150 °C, the decrease in sample quality is attributed to the loss of free water and bound water. SDF began to decompose from 150 °C to 320 °C. Finally, at 600 °C, the mass of the sample was 60.45% of the original mass. The results showed that Jerusalem artichoke SDF had good thermal stability.

Figure 1B shows the results of Fourier transform-infrared spectroscopy (FT-IR). A strong peak appeared at 722 cm^−1^, which proved the existence of –(CH_2_)_n_– (n ≥ 4). The stretching vibration of –OH appeared at 3600 cm^−1^. The peak at 2600 cm^−1^ replaced S–H. The peak near 2350 cm^−1^ represents the characteristic absorption peak of P–H. The characteristic absorption peak at 2100–2200 cm^−1^ is attributed to the C–H stretching vibration of alkynes, and the angular vibration absorption peak of C–H or C–O is in the range of 1300–1400 cm^−1^ [18].

The results of the XRD analysis are shown in Figure 1C. A typical cellulose diffraction peak signal appears near 2θ = 16° (101 crystallographic plane), 23° (002 crystallographic plane), and 35° (040 crystallographic plane), and it can be judged as a cellulose I structure from the peak position. According to the formula, the crystallinity of the sample is 50.21% [19].

The microstructure of Jerusalem artichoke SDF was analyzed, and two scanning electron microscope photographs at 1000 times and 7000 times were obtained, as shown in Figure 1D,E. The typical crystal structure of a cellulose rod can be seen in the electron microscope pictures, which proves that the extracted soluble dietary fiber is in good condition [10].

### 3.2. SDF-Chitosan Film Characterization

#### 3.2.1. Film Thickness Analysis

**Figure 2:**
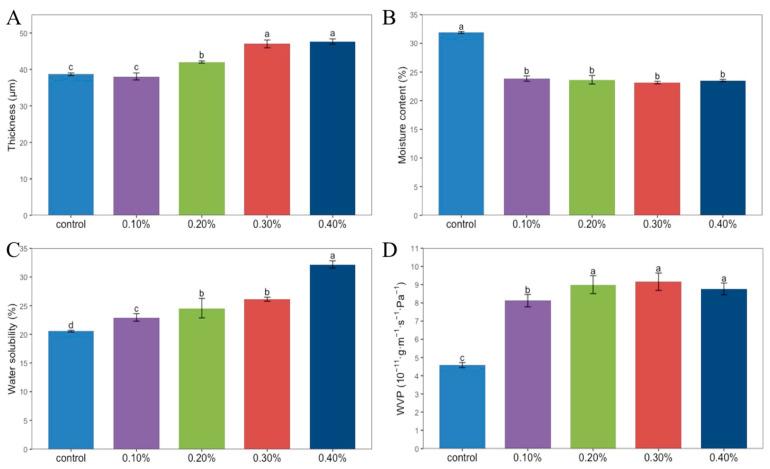
Thickness (**A**), moisture content (**B**), water solubility (**C**), WVP (**D**) of Chitosan films without SDF and with different contents of SDF. Different letters indicate significant differences (*p* < 0.05).

As shown in Figure 2A, the thickness of the films is closely related to the amount of SDF added. With the increase in SDF content from 0.1% to 0.4%, the thickness of the films gradually increased from 38.67 μm to 47.64 μm. Hydroxyl is an important bridge between chitosan molecules. Due to the presence of hydroxyl groups in the SDF, the spacing between chitosan molecules shortened, and the density of the SDF film increased to form a denser structure, so the thickness of the SDF film increased correspondingly.

#### 3.2.2. Analysis of Moisture Content, Water Solubility, and Water Vapor Permeability (WVP)

The moisture content of the films is shown in Figure 2B. The moisture content of the four composite films was about 23%, and the moisture content of the control group was the highest, which was 31.89%. A single factor analysis of variance showed that there was no significant difference among the four concentrations of SDF composite films, while the difference was significant with the control group. It can be seen that the addition of SDF caused a decrease in the moisture content of the film.

It can be seen in Figure 2C that with the increase in SDF content from 0% to 4%, the water solubility of the film increased from 20.55% to 32.19%. With the addition of SDF, the proportion of soluble components in the film also increased [15].

Figure 2D shows the WVP of the film. The WVP of the group without SDF was 4.58 × 10^−11^ g·m^−1^·s^−1^·Pa^−1^, which was significantly lower than that of the four groups with SDF added. The WVP of the film increased first and then decreased with the increase in SDF content. When the SDF content was 0.3%, the WVP of the film was the highest, which was 9.16 × 10^−11^ g·m^−1^·s^−1^·Pa^−1^.

#### 3.2.3. Morphology and Microstructure of Thin Films

**Figure 3:**
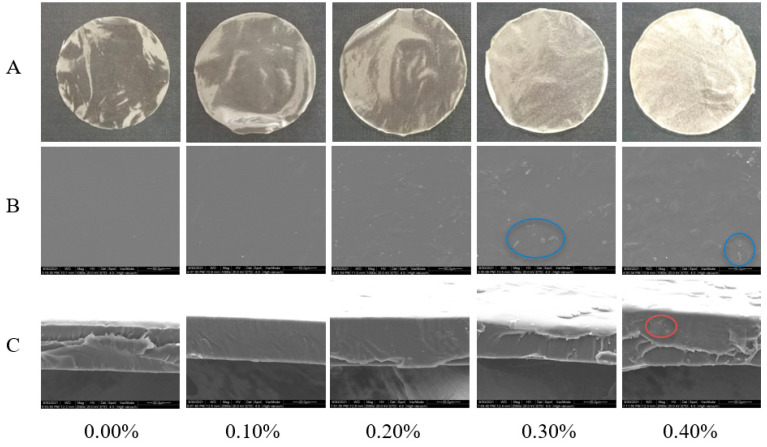
Photographs (**A**), micrographs of surface (**B**) and micrographs of cross section (**C**) of films with different SDF contents. The blue circle marks the graininess of the film surface. The red circle marks the pores in the cross section of the film.

The surface morphology of the films is shown in Figure 3A. The film without SDF was light yellow, smooth, and transparent. After the addition of SDF, the color of the film deepened, the transparency decreased, and the particle sense increased, which were confirmed by the results of the SEM, as shown in Figure 3B. The films with SDF addition levels less than 0.3% had smooth surfaces and no particles. The two kinds of films with SDF contents of 0.3% and 0.4% had granular surfaces. Due to the aggregation of cellulose, the higher the amount of SDF, the higher the roughness of the film surface. Figure 3C shows an SEM image of the cross section of the films. The structure of the films with SDF contents less than 0.4% was continuous and dense without voids and phase separation. Small pores appeared in the film with 0.4% SDF. In addition, with the increase in the amount of SDF, the film gradually appeared to have an uneven thickness [8].

#### 3.2.4. TGA Analysis

**Figure 4:**
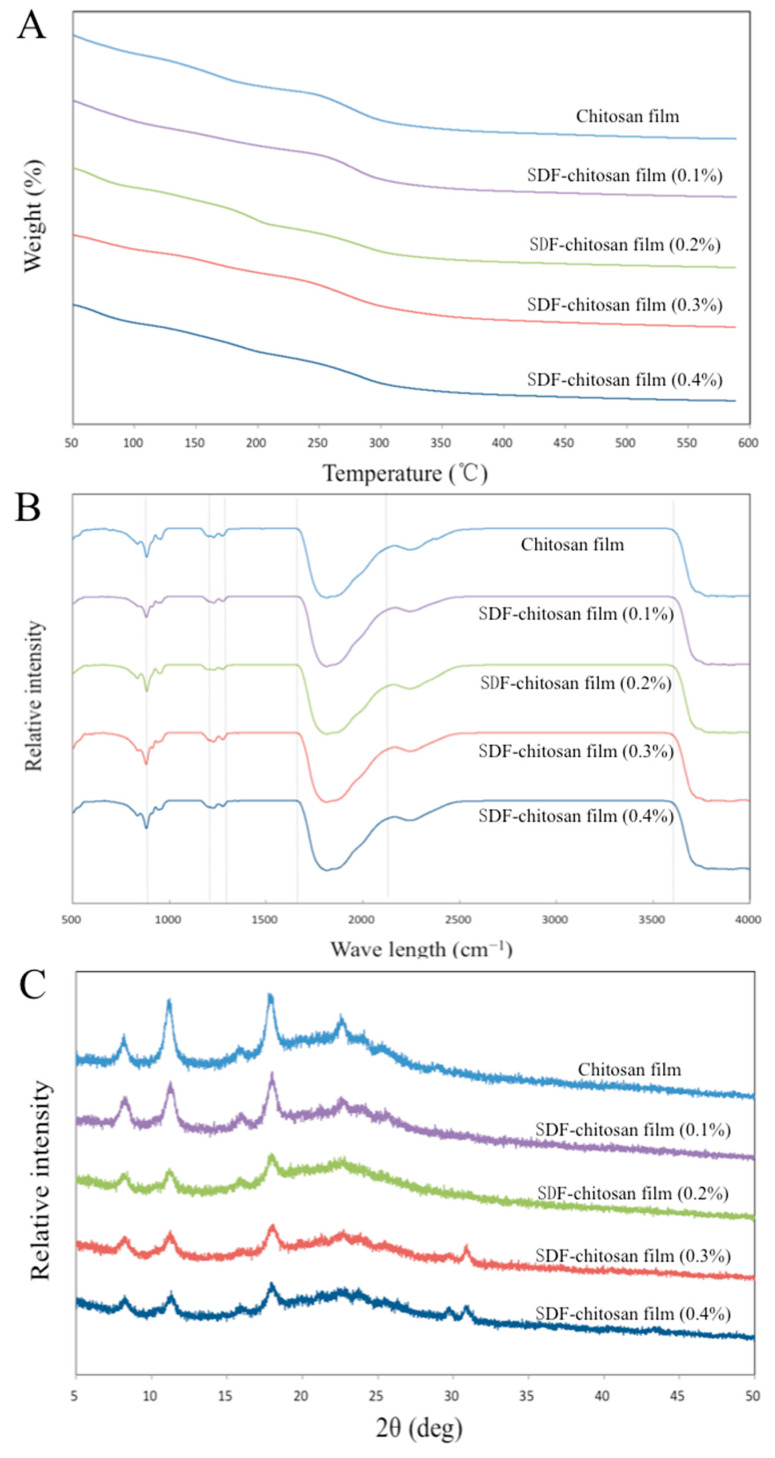
TGA analysis (**A**), FT-IR spectra (**B**), and XRD patterns (**C**) of films.

Figure 4A shows the TGA curves of five films, indicating that the thermal degradation process of the film is divided into three stages. At 50–140 °C, the weight loss was attributed to the loss of free water. The bound water and glycerol in the molecule evaporated at 140–280 °C. At 280–500 °C, the polymer in the film gradually decomposed, and the functional-group-related chain broke. The weight tended to be stable above 500 °C. The residual masses of the five films after the TGA were 17.39%, 25.24%, 23.93%, 30.56%, and 27.00% of the original mass, respectively. It could be seen that the thermal stability of the films with SDF was better than the control group.

#### 3.2.5. FT-IR Analysis

The FT-IR spectra reveal the molecular interactions between different film components, as shown in Figure 4B. The O–C–O stretching vibration appeared at 1640 cm^−1^. The peaks at 931 cm^−1^ and 790 cm^−1^ represent the α-1,6-glycosidic bond and α-1,4-glycosidic bond, which are the main chemical bonds of monosaccharide molecular linkage in cellulose. The stretching vibration of the hydrogen bond (O–H) appeared at 860 cm^−1^ in the fingerprint area, and the infrared absorption appeared at about 3600 cm^−1^ in the characteristic area, which confirmed the existence of a hydrogen bond. The stretching vibrations at 1170 cm^−1^ and 1245 cm^−1^ represent N–H and C–H, respectively [11].

#### 3.2.6. XRD Analysis

As shown in Figure 4C, the XRD analysis revealed the crystalline properties of the films. The spectrum shows that diffraction peaks appeared at 7.99°, 11.04°, 15.34°, 17.74°, and 22.29°, indicating that the films had certain crystallinity. The diffraction peak at 11.04° was related to the 020 diffraction plane of the chitosan anhydrous crystal. However, with the increase in SDF content, the diffraction peak gradually weakened. This could be explained as the formation of hydrogen bonds, which destroys the close packaging between chitosan and cellulose molecules. The existence of hydrogen bonds is discussed in 3.2.5, which supports this view. In addition, the addition of glycerol in the coating-forming solution increased the fluidity of the polymer, with a reduction in the cohesion of the matrix, hindering the arrangement of long chains. The diffraction peaks at 29.41° and 30.57° appeared when the SDF content was 0.3% and 0.4%, which may be caused by the 040 crystal plane of cellulose [20].

### 3.3. Application of Film in Blueberry Preservation

#### 3.3.1. Weight Loss Rate, Decay Rate, and Frost Coverage Index of Blueberries during Storage

**Figure 5:**
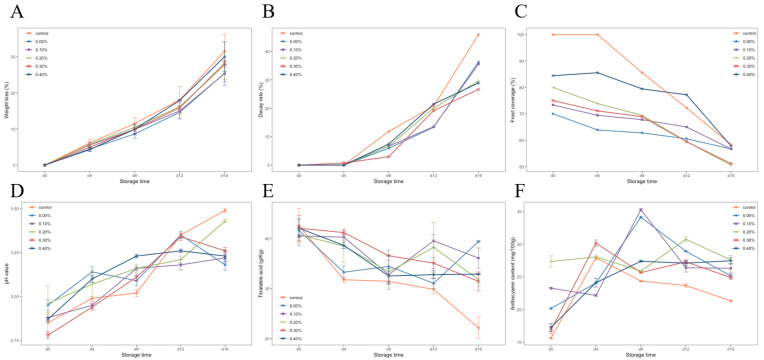
Weight loss rate (**A**), decay rate (**B**), frost cover index (**C**), pH (**D**), titratable acidity (**E**), and anthocyanin content (**F**) of blueberries during storage.

The weight loss rates of blueberries with different treatments during storage are shown in Figure 5A. The weight loss rate of the control group was significantly higher than that of the other groups, and the weight loss rate of the control group reached 31.55% on day 16. It was proven that coating film treatment on blueberries could reduce the weight loss of blueberries. After 16 days of storage, the film with 0.1% SDF showed the best performance in preventing pulp weight loss, and the weight loss rate was 25.45%. With the increase in SDF content, the ability of the film to prevent weight loss decreased.

Figure 5B shows the changes in the decay rates of the blueberries in each group. It can be seen that the decay rate of the blueberries in each group increased, and the decay rate accelerated after 8 days of storage. On the 16th day of storage at room temperature, the highest decay rate was 45.39% in the control group, followed by the no SDF group. The best protection against rot was the 0.3% SDF coating, which reduced the rot rate by 19.26% compared with the control group. The results showed that the coating treatment could slow down the corruption of blueberries, and the addition of SDF enhanced this ability of the coating.

Figure 5C shows the changes in frost covering index of the blueberries in each group during storage. The results showed that, at the beginning of storage, the frost cover index of blueberries treated with a coating was lower than that of the control group. After four days of storage, the frost covering rate of the control group decreased rapidly, while the blueberries treated with a coating were relatively stable. In the treatment group with the 0.4% SDF coating, the change of the frost cover rate was only 26.66% during the whole storage period (41.67% in the control group). This shows that although the coating treatment would lead to the loss of blueberry surface frost, the cellulose composite membrane could play a good protective role on the frost in storage.

#### 3.3.2. The pH, Titratable Acid (TA), and Anthocyanin Content of Blueberries during Storage

The changes in pH and titratable acid in blueberries during storage are shown in Figure 5D,E. With the progress of storage, organic acids in blueberries were gradually consumed, and the pH showed an upward trend, while titratable acid showed a downward trend. It can be seen in the diagram that the pH of blueberries treated with a coating was lower than that of the control group, and the titratable acid was higher than that of the control group. This shows that coating treatment could protect the organic acids in fruits and slow down the consumption of organic acids.

Figure 5F shows the changes in anthocyanin content in blueberries during storage. It can be seen that the anthocyanin content of the blueberries in each group increased and then decreased. The peak of anthocyanin content in the control group and the 0.3% SDF coating group appeared on the fourth day of storage; the peak of anthocyanin content in the 0%, 0.1%, and 0.4% SDF coating groups was delayed to the eighth day of storage. The addition of 0.2% SDF delayed this peak to the 12th day of storage, and the change rate of anthocyanin content in this group was the smallest during the whole storage period. It indicated that SDF coating was beneficial to anthocyanin accumulation in blueberry fruit.

#### 3.3.3. Changes in Blueberry Hardness during Storage

**Figure 6:**
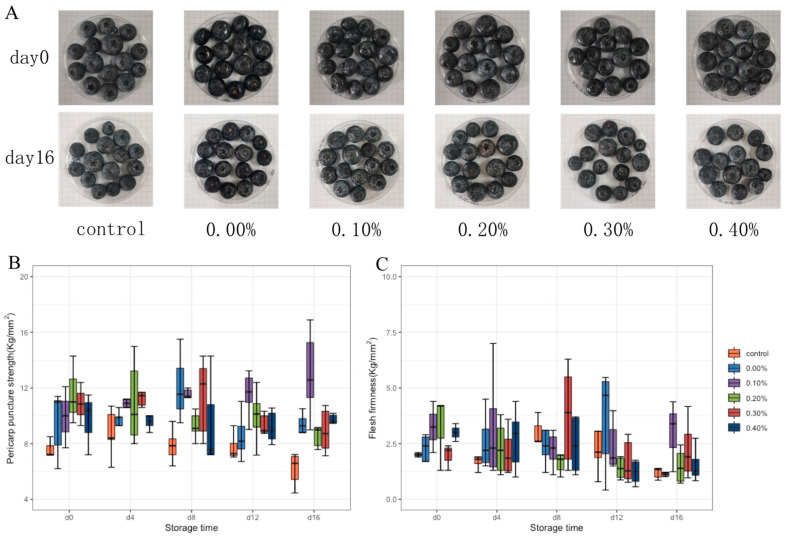
Photographs (**A**), pericarp puncture strength (**B**), and average flesh firmness (**C**) of blueberries during storage.

Pericarp puncture strength and average flesh firmness can accurately reflect fruit firmness. The pericarp puncture strength and average flesh firmness of blueberry were tested by a texture analyzer, and the results are shown in Figure 6B,C. During storage, the pericarp puncture strength and average flesh firmness of blueberries in the control group first increased and then decreased. On the 16th day of storage, compared with the control group and the group without SDF, the coating with SDF showed better performance in the maintenance of fruit hardness. Especially when the SDF content was 0.2%, the fruit could maintain good hardness during 16 days of storage.

## 4. Discussion

In this study, the soluble dietary fiber (SDF) extracted from Jerusalem artichoke was used to prepare a blueberry coating. First, the extracted SDF was characterized, and the results showed that it had a typical cellulose crystal structure. The properties and bioactivity of dietary fiber are related to its complex structure. As a polysaccharide, cellulose is very suitable for membrane materials, but most of the cellulose is insoluble, which increases the difficulty of preparing a coating-forming solution [21]. The FT-IR spectrum of SDF proved the existence of –OH in the sample, which gave Jerusalem artichoke dietary fiber good water solubility and was conducive to the preparation of a coating-forming solution. High-crystalline structures need to consume more energy during thermal decomposition, so materials with high crystallinity generally have higher thermal stability [22]. The results of the XRD analysis showed that the crystallinity of the extracted SDF was as high as 50.21%, and the TGA analysis also showed that the SDF had good thermal stability, which was the required performance of membrane materials [23].

The miscibility of each component in the coating-forming solution affects the microstructure of the film and further affects the mechanical properties and barrier properties of the film. The presence of –OH in SDF enhanced its hydrophilicity, but it was still difficult to dissolve with chitosan. The addition of acetic acid contributes to the dissolution of the two components [24]. In addition, glycerol is hydrophilic, which makes the membrane components form a closer connection by association. When an appropriate amount of SDF was added, the components in the coating-forming solution could effectively dissolve each other, and the prepared film had a dense and uniform microstructure, showing good mechanical properties and barrier properties [25]. The barrier property is a very important performance, which can reflect the preservation ability of a coating. A coating with a good barrier property can effectively prevent microbial infection, reduce fruit moisture loss, and improve fruit storage resistance. If excessive SDF is added into the coating-forming solution, which exceeds the miscible ability of each component, it will cause the separation of membrane components, resulting in an uneven structure of the prepared film and a decrease in the barrier performance [26]. SDF has good thermal stability, and the addition of SDF improves the thermal stability of the film, especially when the SDF content is 0.3%, which is when the thermal stability of the film is the best and most suitable for food packaging [27].

The effect of the coating on blueberry preservation was further tested. The decline in blueberry quality during storage is mainly manifested as weight loss and softening. The loss of blueberry weight is mainly due to the loss of nutrition and water during storage. After weight loss, the skin of the fruit will wrinkle and the luster becomes dark [28]. The blueberry photos in Figure 6A show that the blueberry fruits of all groups were full and glossy on the 0th day. On the 16th day of storage, the blueberries appeared with different degrees of collapse, softening, and peel fold. The film without SDF or with a small amount of SDF (0.1%) had a good ability to prevent fruit weight loss, and this ability decreased with the increase of SDF content [29]. When the SDF content exceeds the mutual solubility of each component in the solution, the microstructure of the film will become uneven and even generate pores. This may be the cause of the increased weight losses of the high-SDF-content groups. Although the excessive addition of SDF resulted in an increase in the weight loss rate of blueberries during storage, it still had a good ability to prevent blueberry corruption, indicating that only water was volatilized through the pores of the film, without the loss of other nutrients in the blueberries [30]. The maturation of the fruit cell wall and the proliferation of microorganisms will cause the softening of blueberry fruit. In the early stage of storage, the peel and pulp of untreated blueberries increased in hardness due to dehydration. In the late stage of storage, the cell wall of the fruit gradually matured and damaged, and the microorganisms began to grow and reproduce, resulting in the softening of the fruit. The coating treatment can effectively delay the cell wall damage. The addition of SDF enhanced the viscosity and support of the coating on blueberries. On the one hand, the coating effectively blocked the invasion of microorganisms from the surface of fruit. On the other hand, it delayed the evaporation of water and the damage of the fruit cell wall, so the coating could maintain the hardness of blueberries and enhance the storage resistance of blueberries [31].

During the storage of blueberries, the consumption of organic acids in the fruit will lead to the growth and reproduction of microorganisms and accelerate corruption. The coating with SDF had a good protective effect on the organic acids in blueberries. Anthocyanins are secondary metabolites in blueberry metabolism [32]. Anthocyanins not only have high nutritional value but also have strong antioxidant capacity and are important nutrients in blueberries. At the beginning of storage, due to the postharvest ripening of blueberries, metabolism produced a certain amount of anthocyanin, and the anthocyanin content in the blueberries increased. With the extension of storage time, anthocyanins were oxidized and consumed. The coating could prevent blueberries from contacting with air and slow down the oxidation of anthocyanins. In addition, the coating treatment slowed down the consumption of organic acids, and the acidic environment was more conducive to the preservation of anthocyanins. In conclusion, the coating with SDF can effectively slow down the loss of nutrients in blueberries during storage [33].

## 5. Conclusions

In this study, soluble dietary fiber (SDF) with high purity and good state and properties was extracted from Jerusalem artichoke residue, and the extracted SDF was characterized. Five concentrations of SDF-chitosan composite coatings were prepared using the extracted SDF. The characterization of the coatings shows that the coatings have excellent properties to apply to blueberry preservation, and the results showed that the coating-added SDF could effectively improve the quality of blueberries during 16 days storage. The SDF coating has potential and broad prospects in application of the preservation of blueberries and other berries. The comprehensive utilization of Jerusalem artichoke residue, reducing the manufacturing cost of coating, and expanding the application range of coating need further research.

## Figures and Tables

**Table 1:** The compositions of the SDF-chitosan coating solutions.

Components	Proportion				
	Control	0.10%	0.20%	0.30%	0.40%
Chitosan (g)	2 g	2 g	2 g	2 g	2 g
Glacial acetic acid (mL)	1 mL	1 mL	1 mL	1 mL	1 mL
Glycerol (mL)	0.6 mL	0.6 mL	0.6 mL	0.6 mL	0.6 mL
SDF (g)	0 g	0.10 g	0.20 g	0.30 g	0.40 g
Distilled water (mL)	100 mL	100 mL	100 mL	100 mL	100 mL

## Data Availability

No new data were created or analyzed in this study. Data sharing is not applicable to this article.
